# Prevalence, Intensity and Psychosocial Burden of Acne Itch: Two Different Cohorts Study

**DOI:** 10.3390/jcm12123997

**Published:** 2023-06-12

**Authors:** Marta Szepietowska, Beata Bień, Piotr K. Krajewski, Aleksandra A. Stefaniak, Łukasz Matusiak

**Affiliations:** Department of Dermatology, Venereology and Allergology, Wroclaw Medical University, 50-367 Wrocław, Polandaleksandraannastefaniak@gmail.com (A.A.S.);

**Keywords:** itch, acne, quality of life, depression, anxiety, burden

## Abstract

Background: Chronic itch is a common symptom of inflammatory skin diseases. This study was undertaken to evaluate the presence and intensity of itching in two different cohorts of acne subjects. Additionally, the influence of itching on the psychosocial status of acne individuals was assessed. Methods: Consecutive acne patients seeking dermatological advice and university students diagnosed with acne during dermatological screening were considered. The clinical and psychological aspects of acne were assessed using a variety of instruments. Results: About 40% of acne subjects in both cohorts reported itching. The mean WI-NRS during the last 3 days in acne patients was 3.83 ± 2.31 points (mild itch) and was significantly more severe (*p* < 0.001) than in university students diagnosed with acne (2.09 ± 1.29 points). Itch intensity did not depend on the clinical severity of acne. In consecutive acne patients, itch intensity correlated with quality-of-life impairments (assessed using DLQI and CADI) and HADS scores. There was no correlation between itch intensity and stigmatization levels. Conclusions: Itching seems to be a common phenomenon in acne sufferers. Acne itch significantly influences patients’ well-being and should be considered in a holistic approach to acne patients.

## 1. Introduction

Acne is a very common chronic inflammatory skin disease that predominantly affects adolescents and young adults. Data from recent decades have documented that acne may also appear in older adults [1,2]. The prevalence of acne among adolescents is about 80% worldwide; however, moderate to severe acne accounts for 20% of all acne sufferers. Adult acne is an increasing problem and is currently estimated to affect 40% of the population [3,4]. Although acne is not a life-threatening disease, it frequently has serious psychosocial consequences [5,6,7,8,9]. Many acne patients suffer from depression and anxiety, and suicidal thoughts and attempts are not uncommon [6]. The quality of life of acne patients is heavily decreased [10]; the visibility of lesions commonly leads to increased levels of stigmatization [11]. The clinical manifestations of acne vary among individuals. Facial acne seems to be the most common; however, in many patients, lesions are located also, or even exclusively, on the trunk [12,13]. The morphology of non-inflammatory and inflammatory acne lesions is well known; however, there is limited data on subjective symptoms. For years, acne has been regarded as a non-itchy skin condition. To the best of our knowledge, there are only a few reports on itching in acne patients available in the literature, indicating that at least some acne patients may experience itching sensations [14,15,16,17,18].

Therefore, the aim of the current study was to assess the prevalence of itch in two different cohorts of subjects suffering from acne. Moreover, the psychosocial burden of acne itching was evaluated.

## 2. Materials and Methods

### 2.1. Subjects

Two cohorts of individuals suffering from acne were studied. The first cohort (Cohort I) consisted of 104 consecutive patients visiting dermatology offices due to acne in 2022. There were 69 (66.3%) females and 35 (33.7%) males with a mean age of 20.38 ± 5.78 years. The mean duration of their acne was assessed as 5.97 ± 4.73 years. Subjects included in the study were newly diagnosed acne patients and had not been previously treated for acne. Only subjects without a previous history of inflammatory dermatoses associated with itching, e.g., atopic dermatitis or psoriasis, were included. The usage of cosmetics before enrolment was allowed. The second cohort (Cohort II) included 213 university students who were diagnosed with acne by experienced dermatologists during dermatology screening. A total of 202 students agreed to participate in the project (94.8% response rate). Cohort II consisted of 127 (62.9%) females and 75 (37.1%) males. The mean age of this study group was 22.7 ± 1.6 years. Similarly to Cohort I, these subjects were not taking any anti-acne medications. Detailed demographics of both cohorts are given in Table 1. The study was approved by the local Ethical Committee of Wroclaw Medical University (KB-663/2022).

### 2.2. Assessment of Acne Severity

Acne severity was evaluated using the Investigators Global Assessment (IGA). This is a validated scoring instrument graded from 0 points (clear skin) to 4 points (severe acne) [19].

### 2.3. Assessment of Itching

The presence of itching was diagnosed based on a single question: “Have you ever had itch within skin area with acne lesions?” The intensity of the itch was measured using a numeral rating scale (NRS) [20]. NRS is an unidimensional scale, where 0 points indicate no itch and 10 points indicate the worst imaginable itch [20]. In both cohorts of subjects, the intensity of the worst itch during the last 3 days was considered. Additionally, in cohort I patients were asked to assess the worst itch intensity during the whole period of their disease. This was not employed for Cohort II, as at least some individuals with almost clear skin referring to minimal acne were not aware of having acne. The following cut-off points were used to interpret NRS: 0 points—no itch, 1–3 points—mild itch, 4–6 points—moderate itch, 7–8 points—severe itch, and at least 9 points—very severe itch [20].

### 2.4. Assessment of Psychosocial Burden

The Dermatology Life Quality Index (DLQI) and Cardiff Acne Disability Index (CADI) were used to assess Quality of Life (QoL) impairment. The DLQI is considered a dermatology-specific instrument and has a recall period of 1 week. It contains 10 items, each with a score of 0 to 3 points (0 points—“not at all”, 1 point—“a little”, 2 points—“a lot”, and 3 points—“very much”. The final scores were summed up and the total score ranged from 0 points to 30 points. The higher the score, the more QoL decreased [21]. CADI is a QoL assessment tool specially designed to evaluate QoL impairment in acne patients. It consists of five questions with a recall period of one month. The score of each answer ranges from 0 points to 3 points. The final score is calculated by summing the scores of each question, which can range from 0 to 15 points. Similarly to the DLQI, the higher the score, the more impaired the QoL [22,23]. The 6-Item Stigmatization Scale (6-ISS) was employed to assess the level of stigmatization. The 6-ISS is an instrument built with 6 questions. The patients answer each question obtaining 0 to 3 points: 0 points—“not at all”, 1 point—“sometimes”, 2 points—“very often”, and 3 points—“always”. The scale ranges from 0 to 18 points, and a higher score indicates a greater perception of stigmatization [24,25]. Depression and anxiety were evaluated only in Cohort I subjects. This was performed using the Hospital Anxiety and Depression Scale (HADS) [26]. HADS is a well-known self-assessment scale consisting of seven questions related to depression and seven questions related to anxiety. Each question is scored from 0 to 3 points, giving a maximum score of 42 points on the whole scale. Both subscales, HADS Depression (HADS-D) and HADS Anxiety (HADS-A), have a maximum score of 21 points. HADS has a recall period of one week. Higher scores indicate greater depression/anxiety levels [26]. All the above-mentioned tools were used in validated Polish language versions [27,28,29].

### 2.5. Statistical Analysis

Statistical analysis was performed using IBM SPSS Statistics v. 26 (SPSS INC., Chicago, IL, USA) software. In the beginning, all the data were assessed for parametric and non-parametric distribution using the Kolmogorov–Smirnov normality test. The minimum, maximum, mean, standard deviations, and ranges were calculated. The Student’s T test or Mann–Whitney U test for parametric and non-parametric data were employed for quantitative data. Depending on normality, Spearman’s and Pearson’s correlations were used for the correlation assessments. Qualitative data were analyzed using the Chi2 test. Differences in analyzed data between more than two groups were evaluated, depending on normality, using ANOVA or Kruskal–Wallis 1-way analysis of variance on ranks. A 2-sided *p* value less than 0.05 was statistically significant.

## 3. Results

### 3.1. Cohort I Results

The mean IGA for acne severity in this group of patients was 3.22 ± 0.7 points. The majority of the subjects, 49 (47.1%) patients, suffered from moderate acne. A total of 39 (37.5%) patients had severe acne, and in the remaining 16 (15.4%) subjects, mild acne was diagnosed. Acne was significantly more severe (*p* = 0.012) among males than females (Table 1).

Itching during the entire acne period was reported by 41 patients (39.4%). The same group of patients experienced itching during the last 3 days. More females (31, 44.9%) reported itching than males (10, 28.6%); however, the observed difference did not reach statistical significance (*p* = 0.107). The mean worst itch intensity during the acne period was assessed as 3.83 ± 2.31 points, indicating an itch of mild intensity. There was no significant difference in itch intensity for the entire acne period between females and males (3.55 ± 2.20 points and 5.10 ± 2.75 points, respectively). For the whole study group, the mean worst itch NRS score of the last 3 days was 3.12 ± 2.28 points. Males reported significantly (*p* = 0.004) more severe itching during the last 3 days than females (4.77 ± 2.54 points and 2.48 ± 1.73 points, respectively) (Table 2).

The intensity of itch (both for the entire acne period and for the last 3 days) did not correlate with the clinical acne severity assessed using IGA. However, itch intensity showed a positive correlation with DLQI (r = 0.394, *p* = 0.011 for the entire disease period; and r = 0.447, *p* = 0.011 for itching during the last 3 days). Additionally, itch intensity assessed for the entire acne period and in the last 3 days correlated positively with CADI (r = 0.415, *p* = 0.02; and r = 0.373, *p* = 0.039, respectively) in female groups (Table 3). There was no relationship between itch intensity and stigmatization levels. Itch intensity for the entire disease period and for the last 3 days was significantly correlated with the total HADS scores (r = 0.482, *p* = 0.002; and r = 0.390, *p* = 0.002, respectively). Similar relationships were observed for the HADS-D scores (r = 0.427, *p* = 0.006; and r = 0.352, *p* = 0.026, respectively). Interestingly, only itch severity during the entire disease showed a positive correlation with the HADS-A results (r = 0.325, *p* = 0.041) (Table 3).

### 3.2. Cohort II Results

According to IGA, the mean clinical disease severity was assessed as 1.92 ± 0.74 points with no difference between females and males (1.90 ± 0.78 and 1.95 ± 0.68, respectively) (Table 1). A total of 91 out of 202 subjects (45.0%) were diagnosed with mild acne, 64 (31.7%) individuals suffered from minimal acne (almost clear skin), and the remaining 47 (23.3%) patients were diagnosed with mild acne. There were no subjects suffering from moderate or severe acne. Comparing both cohorts, patients from Cohort I had significantly more severe acne (*p* < 0.001) than those from Cohort II. This was also noted when comparing acne severity separately in females and males from the two cohorts of subjects (Table 1).

In Cohort II, itching was found in 81 (40.1%) subjects. There was no difference in the itch prevalence between females and males, which were 52 (40.9%) and 29 (38.7%), respectively. The mean worst itch intensity during the last 3 days for the whole group was 2.09 ± 1.29 points, which was similar for females and males (2.04 ± 1.33 points and 2.17 ± 1.23 points, respectively) (Table 4).

Itching was significantly lower (*p* < 0.001) in comparison to the mean itch intensity in Cohort I patients (3.12 ± 2.28 points). Similarly, in Cohort I, itch intensity during the last 3 days did not correlate with acne severity (IGA) or stigmatization level (6-ISS). However, in contrast to patients from Cohort I, itch intensity in Cohort II individuals did not show any significant relationship with QoL scores, as assessed by both DLQI and CADI.

## 4. Discussion

Itching is defined as an unpleasant sensation leading to scratching [30,31,32]. Acute itching lasting less than 6 weeks is usually considered a defense mechanism. Chronic itching with a duration longer than 6 weeks is frequently associated with long-term medical problems [32]. Chronic itching is a common phenomenon in the general population. In a population-based study, the 12-month cumulative incidence of chronic itching was estimated to be 7% and it increased with age [33]. According to the International Forum for the Study of Itch (IFSI), there are six itching categories: dermatological (occurring in skin diseases), systemic, neurological, somatoform (psychogenic), itch of mixed origin, and itch of undetermined origin [32]. Itching can be localized or generalized. It is suggested that localized itching is mainly caused by itchy dermatosis, and its location is usually limited to the presence of inflammatory skin lesions [31]. Itching is the most common subjective symptom in dermatology. It can occur under different skin conditions. Skin infections and infestations are very commonly associated with itching, but also many inflammatory skin diseases, such as atopic dermatitis, seborrheic dermatitis, psoriasis, or lichen planus, are highly itchy dermatoses [30,31]. Chronic itching is a devastating symptom [34,35]. It negatively influences the psychosocial status of sufferers and is linked to an increased risk of the development of depression and anxiety [34,35]. From the patient’s perspective, itching is frequently regarded as the most important symptom of acne. This was clearly documented in patients with psoriasis, where itching was not only the most bothersome but psoriatic patients also considered itching as the most important factor influencing disease severity [36]. Therefore, it is clear that itch management should be seriously taken into account in the holistic approach to itchy subjects, including those with itchy dermatoses. Studies dealing with an itch in various diseases are of great help in extending knowledge on itch pathogenesis and clinics, and they significantly contribute to a better understanding of this pathological symptom, which may have an effect on itch management in the future.

Many studies on itching in various inflammatory skin diseases, such as atopic dermatitis, lichen planus, and psoriasis, have been conducted [37,38,39,40,41,42]. However, aspects of itching in acne vulgaris have raised the attention of a limited number of researchers, and the literature data on acne itch are scarce [14,15,17]. In the current study, we decided to evaluate the prevalence and intensity of an acne itch in two very different groups of acne patients. Consecutive patients seeking advice because of their acne and showing up for acne management in dermatology outpatient units were grouped as Cohort I. Cohort II was recruited during the population-based dermatological screening of university students. Subjects diagnosed with acne by a dermatologist were invited to participate in the project. Therefore, it is not surprising that both cohorts differed in the context of acne severity. Cohort I represented patients with more severe acne, hoping for effective acne treatment. In Cohort II, there were only individuals with minimal and mild acne, and some of them did not even consider themselves as having acne lesions. Having studied two different populations, we aimed to obtain a better overview of itch prevalence and intensity in patients with acne. It is well-known that drugs, both topical and systemic, may contribute to the development and exacerbation of itching sensations [31,43]. It is worth mentioning that we did our best to eliminate this factor, as dermatology outpatients were only included in the study if they were not ever treated for acne. All subjects in Cohort II did not receive any anti-acne medications before taking part in the study.

Itching in both cohorts was diagnosed in almost the same percentage of subjects: 39.4% in Cohort I and 40.1% in Cohort II, respectively. This may suggest that having acne, independently of its severity, is a sufficient factor leading to the creation of an itching sensation. However, although we were unable to find significant correlations between itching and acne severity, itch intensity in patients visiting dermatologists due to their acne was significantly higher than that in the population-based study group. It is obvious that acne in Cohort I was considered a problem by the patients (who were seeking medical help), and skin lesions could have influenced the psychological status of this group of subjects. It is not possible to completely exclude that this situation, different from Cohort II, was the factor influencing the intensity of the itch. It is well known that psychological stress has a negative impact on itch intensity [34,44]. Our results on the prevalence and intensity of acne itching are in agreement with some data reported previously. Reich et al. [14] evaluated 108 teenagers with acne and found itching at the time of examination in 13.9% of subjects, and a further 36.1% reported itching in acne lesions in the past. Similarly, a study of 371 Chinese acne patients showed that itching was present in 45% of males and 46% of females [16]. Additionally, Huet et al. [18] found itching in 48.55% of patients with acne. A higher frequency of itching was reported by Lim et al. [15], who studied 120 acne patients with a mean age of 22 years (age range: 12–40 years), and demonstrated that 70% of the patients reported itching in acne lesions either at the time of study performance or in the past. However, we were unable to find any differences in the itch prevalence in patients with non-inflammatory and inflammatory acne lesions. It is worth mentioning that some of the patients were under anti-acne treatment, which may increase the incidence of itching sensations. An interesting questionnaire-based study on acne itching was conducted among students in Norway [17]. Based on 493 subjects who self-reported having acne lesions, the authors documented itching in 13.8% of subjects reporting ‘a lot of acne’ and in 29.9% of subjects reporting ‘very much acne’ [17]. However, in our opinion, these results should be treated with caution, as the respondents answered the following general questions concerning itching: ‘During the last week, did you have itchy skin?’. This means that the location of the itch was not defined and that itching was not specifically linked with acne lesions.

Concerning itch intensity during the last 3 days, our patients with acne (Cohort I) reported mild itching (NRS: 3.12 ± 2.28 points); however, itch intensity in males (NRS: 4.77 ± 2.25 points) was significantly more severe than in females (NRS: 2.48 ± 1.73 points) and fulfilled the criteria for itching of moderate intensity. In Cohort II, itching in both females and males was of lower intensity than in Cohort I, was of similar intensity in both sexes and was classified as mild itch. Additionally, Reich et al. [14] reported mild itching (Visual Analog Scale—VAS: 3.1 ± 1.9 points) at the time of examination in their acne group without differences in itch intensity between females and males. Similar observations of mild itching at the time of examination of acne subjects (VAS: 1.2 ± 1.6 points) were documented by Lim et al. [15]. VAS was also used to evaluate itch intensity during the last 24 h in acne patients from China. Both females and males suffered from mild itch (1.34 ± 0.12 points and 1.28 ± 0.14 points, respectively) [16]. A limitation of the above-mentioned result is that the readers are not aware of what kind of itching—average or worst—was assessed by the authors.

We were unable to find a correlation between itch intensity and clinical severity of acne. Similar observations were made by others [14]. The only study suggesting a relationship between the severity of acne and itching was a Norwegian study. The prevalence of itch was higher among subjects who self-reported more severe acne [17].

It has been well documented that chronic itching has serious consequences on patients’ psyche [34]. To the best of our knowledge, this is the first study to clearly show that acne itching, even of mild intensity, is positively correlated with QoL impairment. Previous studies by our group showed correlations between itch intensity and QoL assessment in various dermatoses, including psoriasis [44,45,46], atopic dermatitis [47], hidradenitis suppurativa [48], dermatophytosis [49], and basal cell carcinoma [50]. The same correlations were noted between systemic itch and QoL in patients suffering from polycythemia vera [51], chronic kidney disease [52,53], diabetes [54,55], and even in renal transplant recipients [56]. Moreover, in our acne patients, itch intensity correlated with HADS scores. Lim at al. [15] also noticed that 25% of subjects with acne itching reported feeling depressed, and itching influenced mood changes in 55% of acne patients. Additionally, 41% of acne individuals with itching had problems with concentration and 21% had their sleep affected, causing problems both with difficulty falling asleep and with frequent awakenings [15]. In a multicenter study conducted in 13 European countries, the association between the presence of itching in various dermatoses, clinical depression, and suicidal ideation has been well documented [35]. Additionally, itch intensity contributes to the severity of depressive and anxiety reactions in numerous dermatosis and systemic conditions [45,47,54,56,57].

## 5. Conclusions

In conclusion, the results of our study have demonstrated that itching is a common phenomenon in patients with acne. It is usually of mild intensity. Most likely, the intensity of acne itching does not depend on acne severity. However, itching contributes substantially to the psychosocial burden of acne patients. This clearly points to the necessity of considering itch in a holistic approach to acne subjects.

## Figures and Tables

**Table 1:** Demographics of the studied acne cohorts.

	Cohort I	Cohort II	*p*
Subjects [N (%)]			
Whole group	104 (100)	202 (100)	
Females	69 (66.3)	127 (62.9)	NS
Males	35 (33.7)	75 (37.1)	
Age [Mean, range]			
Whole group	20.38 ± 5.78; 12–39	22.7 ± 1.6; 20–31	*p* < 0.001
Females	21.07 ± 6.25; 12–39	22.83 ± 1.85; 21–31	*p* < 0.001
Males	19.0 ± 4.5; 13–32	22.49 ± 1.17; 20–27	*p* < 0.001
Acne severity (IGA) [points]			
Whole group	3.22 ± 0.7	1.92 ± 0.74	*p* < 0.001
Females	3.10 ± 0.71 *	1.90 ± 0.78	*p* < 0.001
Males	3.46 ± 0.61 *	1.95 ± 0.68	*p* < 0.001

IGA—Investigator’s Global Assessment; n—number of participants; NS—non-significant; *—*p* < 0.05.

**Table 2:** Itching in consecutive acne patients (Cohort I).

Itch	Whole Group (*n* = 104)	Females (*n* = 69)	Males (*n* = 35)	*p*
Presence of itching [N (%)]	41 (39.4)	52 (40.9)	10 (28.6)	NS
Mean itch during the entire acne period [points]	3.83 ± 2.31	3.55 ± 2.20	5.10 ± 2.75	NS
Mean itch during the last 3 days [points]	3.12 ± 2.28	2.48 ± 1.73	4.77 ± 2.25	*p* = 0.004

**Table 3:** Correlation between itch intensity and acne clinical severity, along with psychosocial parameters.

Itch	IGA	DLQI	CADI	6-ISS	HADS Total	HADS A	HADS D
During the entire disease							
Whole group (*n* = 41)	NS	r = 0.394 *p* = 0.011	NS	NS	r = 0.482 *p* = 0.002	r = 0.325 *p* = 0.041	r = 0.427 *p* = 0.006
Females (*n* = 31)	NS	r = 0.534 *p* = 0.003	r = 0.415 *p* = 0.02	NS	NS	NS	r = 0.372 *p* = 0.04
Males (*n* = 10)	NS	NS	NS	NS	NS	NS	NS
During the last 3 days							
Whole group (*n* = 41)	NS	r = 0.447 *p* = 0.011	NS	NS	r = 0.390 *p* = 0.002	NS	r = 0.352 *p* = 0.026
Females (*n* = 31)	NS	r = 0.543 *p* = 0.002	r = 0.373 *p* = 0.039	NS	NS	NS	NS
Males (*n* = 10)	NS	r = 0.543 *p* = 0.002	NS	NS	NS	NS	NS

IGA—Investigator Global Assessment; DLQI—Dermatology Life Quality Index; CADI—Cardiff Acne Disability Index; 6-ISS—6 Item Stigmatization Scale; HADS—Hospital Anxiety and Depression Scale; *n*—number of patients; NS—not significant.

**Table 4:** Itching in university students (population-based study).

Itch	Whole Group (*n* = 202)	Females (*n* = 127)	Males (*n* = 75)	*p*
Presence of itching [N (%)]	81 (40.1)	52 (40.9)	29 (38.7)	NS
Mean itch during the last 3 days [points]	2.09 ±1.29	2.04 ±1.33	2.17 ± 1.23	NS

## Data Availability

Data are available upon request.
